# Targeted and Suspect Fatty Acid Profiling of Royal Jelly by Liquid Chromatography—High Resolution Mass Spectrometry

**DOI:** 10.3390/biom13030424

**Published:** 2023-02-23

**Authors:** Christiana Mantzourani, Maroula G. Kokotou

**Affiliations:** Laboratory of Chemistry, Department of Food Science and Human Nutrition, Agricultural University of Athens, 11855 Athens, Greece

**Keywords:** fatty acids, high resolution mass spectrometry, LC–HRMS, royal jelly, suspect analysis

## Abstract

Royal jelly (RJ) is a bee product produced by the mandibular and hypopharyngeal glands of worker honeybees which has attracted special attention because of its numerous pharmacological activities and its applications to dermatology and cosmetics. In 2020, we demonstrated a liquid chromatography–high resolution mass spectrometry (LC–HRMS) method for the determination of seven medium-chain FFAs in RJ samples. The aim of the present work was to extend our studies on FA profiling of RJ, exploring the presence of common long-chain saturated, mono-unsaturated and poly-unsaturated free FAs in RJ samples using this LC–HRMS method. Among twenty common FAs studied by a targeted approach, palmitic acid, stearic acid and oleic acid were found at concentrations higher than the rest of the FAs (the concentrations of these three acids ranged from 37.4 to 48.0, from 17.7 to 24.0 and from 9.4 to 11.1 mg/100 g of fresh RJ, respectively). The high mass accuracy of LC–HRMS allowed the application of a suspect approach, which enabled the exploration of various C9 and C11 FAs, as well as hydroxylated C12 FAs. Nonenoic acid was indicated as the most abundant among these acids. In addition, for the first time, the presence of a variety of regio-isomers of hydroxymyristic, hydroxypalmitic and hydroxystearic acids was demonstrated in RJ samples.

## 1. Introduction

While honey is the most commonly consumed bee product with high nutritional value, honeybees make a variety of other different products, including bee pollen, propolis, bee bread, beeswax and royal jelly (RJ). These natural substances have several uses, ranging from traditional medicine and dietary supplements to dermatology and cosmetics. In the last two decades, scientists have extensively studied these products due to their confirmed beneficial effect on human health [1]. RJ is a yellowish-white material excreted by the mandibular and hypopharyngeal glands of worker honeybees (*Apis mellifera* L.), produced for the nutrition of the queen bee and young larvae in the colony [1,2].

Fresh RJ consists mainly of water (50–70%), followed by proteins (9–18%), carbohydrates (7–18%), fatty acids (FAs) and lipids (3–8%) and small quantities of mineral salts, polyphenols and vitamins [3]. The fluctuation in values reported by different authors can be attributed to the heterogeneous nature of RJ and to the fact that samples have been taken from different locations at different production times. Especially sugar and lipid contents may depend on multiple factors (season, location, botanical origin and others) [4,5].

Normally, the FAs of RJ contain 8-12 carbon atoms (medium chains), and they are usually dicarboxylic or hydroxy FAs (HFAs) terminally or internally hydroxylated and dehydroxylated and either saturated or mono-unsaturated at the 2-position. The major FA of RJ is trans-10-hydroxy-2-decenoic acid (10-HDA), and its content has been adopted as a marker that reflects the quality and authenticity of RJ [6]. Other FAs that have been reported to be present in smaller quantities are 10-hydroxydecanoic, 9-hydroxydecanoic, 7- and 8-hydroxyoctanoic, 9-hydroxy-2-decenoic, 3-hydroxydecanoic, 3,10-dihydroxydecanoic acid, 2-decenoic and octanoic acid [1,7,8]. Different pollen feeding and floral sources can significantly affect the abundance of RJ metabolites. For example, it has been reported that, as a result of different pollen feeding, 10-HDA contents can vary while still meeting the requirement of the international standard of RJ [9,10]. Interestingly, studies with deuterated substrates have revealed that the hydroxylation of FAs at the ω-position is likely catalyzed by P450 enzymes [11].

RJ has received special attention due to its numerous pharmacological activities and has been an attractive ingredient for many applications. Some of its biological activities have been attributed to its bioactive FA components, in particular to 10-HDA [12,13]. For instance, RJ has displayed antitumor activity against Lewis lung carcinoma and colorectal adenocarcinoma cells [14], antimicrobial activity against different bacteria [15], neuroprotective effects [16] and antioxidant and hepatoprotective activities [17]. Additionally, RJ has shown anti-inflammatory activity in different diseases, where 10-HDA has been demonstrated to have the strongest in vitro anti-inflammatory effect [18]. Recently, RJ has been reported to possess estrogen-like activity, attributed to RJ-derived FAs, namely 10-HDA, 3,10-dihydroxydecanoic acid and sebacic acid [19].

At first, HPLC or UPLC methods, as well as capillary zone electrophoresis (CZE), were used for the determination of 10-HDA [20,21,22]. However, the major FAs of RJ are usually determined by gas chromatography–flame ionization detection (GC–FID) or gas chromatography–mass spectrometry (GC–MS), requiring the conversion of free FAs (FFAs) into the corresponding trimethylsilyl derivatives [8,23]. Isidorov et al. have characterized various organic acids and other volatile components of RJ extracts by GC–MS [24]. Moreover, Ferioli et al. have compared the lipid content, FA profile and sterol composition in local Italian and commercial RJ samples using GC–MS [25]. In addition, Ibrahim et al. carried out untargeted and targeted FA profiling of different RJ products based on HPTLC–image analysis [26], and most recently, Ma et al. employed UHPLC–HRMS-based metabolomics for the detailed profiling of RJ metabolites without a derivatization step [10]. Yan et al. recently reported the investigation of the RJ lipidomic profile from different botanical origins combining ultra-high-pressure liquid chromatography/ion mobility-quadrupole-time-of-flight-mass spectrometry (UHPLC-IM-Q-TOF-MS) with gas chromatography–mass spectrometry (GC-MS) [27].

In 2020, we demonstrated a liquid chromatography–high resolution mass spectrometry (LC-HRMS) method for the determination of seven medium-chain FFAs in RJ samples, mainly originating from Greece [28]. Studies on the common FAs (long-chain saturated and unsaturated FAs) in RJ samples are very limited [8,27]. Thus, the aim of our present work was to extend our study on the FA profiling of RJ samples, exploring the presence of common long-chain saturated, mono-unsaturated and poly-unsaturated FFAs. In addition, the presence of various medium-chain FAs and hydroxylated FAs was investigated by the adoption of a suspect analysis LC-HRMS approach. LC-HRMS also offers the possibility of carrying out a suspect search for hydroxylated long-chain FAs in RJ samples, since such a study does not exist in the literature, in contrast to studies on hydroxylated medium-chain FAs.

## 2. Materials and Methods

### 2.1. Chemicals and Reagents

All solvents used were of LC–MS analytical grade. Acetonitrile was purchased from Carlo Erba (Val De Reuil, France), isopropanol and methanol from Fisher Scientific (Laughborough, UK) and formic acid 98–100% from Chem-Lab (Zedelgem, Belgium). Lauric acid was purchased from Acros Organics (>99%), myristic acid from Sigma Aldrich (>99.5%), myristoleic acid from Sigma Aldrich (>99%), pentadecanoic acid from Sigma Aldrich (>99%), palmitic acid from Fluka (analytical standard), 9-palmitoleic acid from Fluka (analytical standard), margaric acid from Sigma Aldrich (>98%), 10-*Z*-heptadecenoic acid from Cayman Chemical Company (>98%), stearic acid from Fluka (analytical standard), oleic acid from Fluka (analytical standard), linoleic acid from Sigma Aldrich (>99%), linolenic acid from Sigma Aldrich (>99%), arachidic acid from Cayman Chemical Company (>98%), bihomo-γ-linolenic acid from Cayman Chemical Company (>98%), arachidonic acid from Sigma Aldrich (>99), 5,8,11,14,17-*cis*-eicosapentanoic acid from Fluka (analytical standard), 7,10,13,16,19-*cis*-docosapentaenoic acid from Cayman Chemical Company (>98%), 4,7,10,13,16,19-*cis*-docosahexaenoic acid from Sigma Aldrich (>98%) and lignoceric acid from Cayman Chemical Company (>98%).

### 2.2. Stock and Working Solutions

Stock solutions of the standard compounds were prepared in a concentration of 1000 mg/L in methanol and stored at 4 °C. Working standard solutions were prepared daily by appropriate dilution. The stability of the standard solution of the compounds (500 μg/L) was examined within 30 days at 4 °C. The retention times and the peak areas of the analytes were not changed for the freshly prepared standard solutions that were kept in the fridge.

### 2.3. Instrumentation

LC–MS/MS measurements were performed using an ABSciex Triple TOF 4600 combined with a micro-LC Eksigent and an autosampler set at 5 °C and a thermostated column compartment. Electrospray ionization (ESI) in negative mode was used for the MS experiments. The data acquisition method consisted of a TOF-MS full scan *m*/*z* 50–850 and several IDA-TOF-MS/MS (Information Dependent Acquisition) product ion scans using 40 V Collision Energy (CE) with 15 V (Collision Energy Spread) CES used for each candidate ion in each data acquisition cycle (1091). This workflow allows quantitation (using TOF-MS primarily) and confirmation (using IDA-TOF-MS/MS) in a single run. Halo C18 2.7 μm, 90 Å, 0.5 × 50 mm^2^ from Eksigent was used as a column, and the mobile phase consisted of a gradient (A: acetonitrile/0.01% formic acid/isopropanol 80/20 *v*/*v*; B: H_2_O/0.01% formic acid). The elution gradient adopted started with 5% of phase B for 0.5 min, gradually increasing to 98% in the next 7.5 min. These conditions were kept constant for 0.5 min, and then the initial conditions (95% solvent B, 5% solvent A) were restored within 0.1 min to re-equilibrate the column for 1.5 min for the next injection (flow rate 55 µL/min). The data acquisition was carried out with MultiQuant 3.0.2 and PeakView 2.1 from AB SCIEX.

EICs were obtained with the use of MultiQuant 3.0.2, which creates the base peak chromatograms for the masses. These chromatograms achieve a mass accuracy window of 5 ppm. The relative tolerance of the retention time window was set lower than ±0.2 min.

### 2.4. Sample Preparation

For the sample preparation, a solid– liquid extraction protocol, previously suggested by Ferioli et al. [25], was employed. An amount of 0.1 g RJ was weighed, and the lipid extraction was performed at room temperature by adding 3 mL of diethyl ether/isopropanol 50/1 (*v*/*v*), stirring for about 30 s every 10 min for an overall period of 30 min and then centrifuging at 4000 rpm for 5 min. The supernatant was collected in a glass tube, and the extraction procedure was repeated twice more. The combined organic extracts were dried over anhydrous Na_2_SO_4_, and, after filtration, the solvent was removed under argon. The dry residue was dissolved in 1 mL of methanol. For the quantification, a 100-fold dilution was performed. LC–MS/MS analysis followed, as described above.

### 2.5. Method Validation

#### 2.5.1. Linearity and Sensitivity

In the present study, 20 saturated and unsaturated FAs were determined using reference compounds. The full list of analytes together with their exact masses [M-H]^-^ and their chromatographic retention times R_t_ are summarized in Table S1 (Supplementary Materials). Limits of detection (LOD) and quantification (LOQ) are also presented in Table S1 [29].

#### 2.5.2. Precision and Accuracy

The EU Commission decision 202/657/EC was followed as a guideline to verify the accuracy and precision of the method. RJ samples were spiked at 500 μg/mL to estimate the recovery and the intra-day variations. The recovery was used for the quantification of the selected compounds in RJ samples. The precision was investigated by means of the relative standard deviation (%RSD).

### 2.6. Royal Jelly Samples

Seven brand products of RJ were collected from the local market in Athens, Greece. Six of them were Greek products, and one was a French product.

## 3. Results and Discussion

### 3.1. Accuracy and Precision Data

RJ samples were spiked with the analytes at 500 μg/mL (three replicates). Accuracy and precision data are summarized in Supplementary Materials Table S1. The recoveries of the analytes ranged from 84 to 100 and the %RSD values ranged from 4.29 to 15.66, depending on the FA.

### 3.2. Analysis of RJ Samples

In our previous work [28], we developed a LC–HRMS method for the determination of FFAs in RJ. The method was applied in the direct quantification of seven medium-chain FFAs of RJ, where the major RJ FA 10-HDA was found to vary from 0.771 to 0.928 g/100 g fresh RJ. The second most abundant FA was 10-hydroxydecanoic acid at concentrations varying from 0.285 to 0.366 g/100 g of fresh RJ, while the other five components were estimated at concentrations lower than 0.100 g/100 g of fresh RJ [28]. In order to expand our method and get a better insight into the lipidomic profile of RJ, in the present work, we study twenty additional common FAs, saturated as well as mono-unsaturated and polyunsaturated.

**Figure 1:**
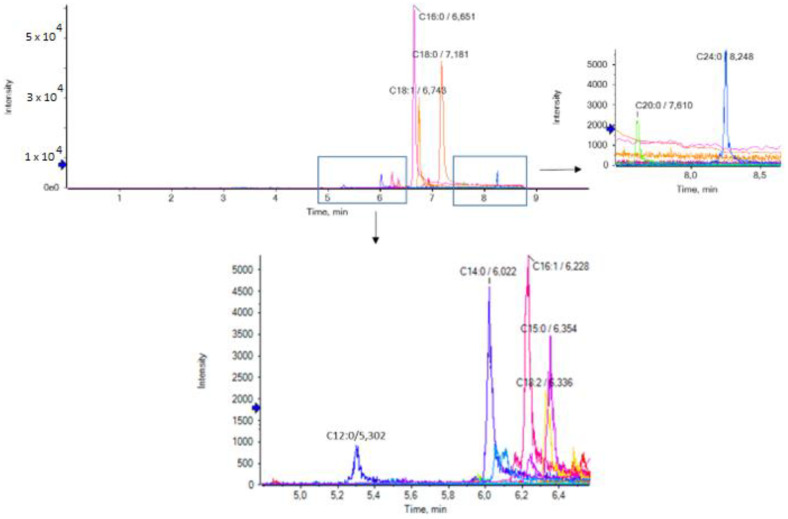
Extracted ion chromatograms (EICs) of the analytes in a representative RJ sample.

The list of the standard compounds along with the exact masses of the deprotonated molecules (theoretical) and the retention time (min) are summarized in Table S1. The extracted ion chromatograms (EICs) of the analytes in a RJ sample are presented in Figure 1 and in Supplementary Material (Figure S3).

The contents of common long-chain FAs in seven RJ extracts are summarized in Table 1 and are expressed as mg of FA per 100 g of fresh RJ. The saturated long-chain palmitic (C16:0) and stearic (C18:0) acids were found to be the predominant FAs in all samples. C16:0 content ranged from 37.4 to 48.0 mg/100 g fresh RJ, while C18:0 content varied from 17.7 to 24.0 mg/100 g of fresh RJ. Oleic acid (C18:1) was the most abundant among the unsaturated FAs, and its content was lower than C16:0 and C18:0, varying from 9.4 to 11.1 mg/100 g of fresh RJ. These three FAs were present at considerably higher concentrations than the rest of the FAs.

**Figure 2:**
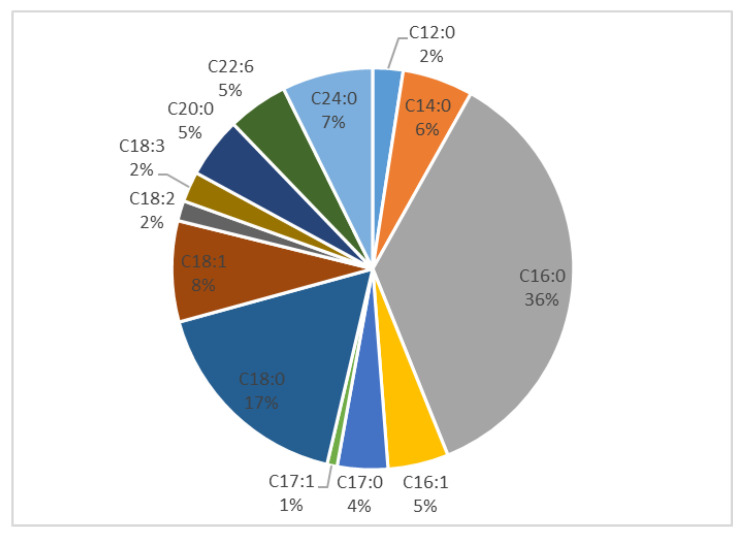
Relative average contents of RJ common long-chain FAs.

The saturated FAs lauric (C12:0), myristic (C14:0), margaric (C17:0) and arachidic acid (C20:0) were found at lower concentrations, ranging from 2.3 to 3.1, from 4.8 to 8.4, from 4.3 to 6.4 and from 4.5 to 7.0 mg/100 g of fresh RJ, respectively. Among the unsaturated FAs, oleic acid was followed by *cis*-9-palmitoleic acid (C16:1, from 5.3 to 6.0 mg/100 g of fresh RJ). Linolenic acid (C18:3), linoleic acid (C18:2) and 10-*Z*-heptadecenoic acid (C17:1) were present in lower quantities, from 1.6 to 4.6, from 1.3 to 1.7 and from 1.0 to 1.4 mg/100 g of fresh RJ, respectively. C20 and C22 poly-unsaturated FAs with 3 or more double bonds were either not detected or were present in trace amounts. Figure 2 shows the relative contents of common long-chain FAs found in RJ samples.

### 3.3. Suspect Analysis

The high mass accuracy of HRMS detection offers the advantage of carrying out non-targeted or suspect analysis in real samples when standards are not commercially available or are missing. Thus, targeted analysis based on reference standards may be combined with non-targeted or suspect analysis in a single run of LC-HRMS. Such approaches have been used in foods, permitting detection and identification of unknown or suspect components [28,30,31,32].

To extend our studies on FA profiling of RJ, we adopted such approaches to explore the existence of various medium-chain saturated or unsaturated as well as hydroxylated FAs. More specifically, since we have previously studied C8:0 and C10:0 FAs [28], we first screened our RJ samples for C9:0 FAs (nonanoic, nonenoic, hydroxynonanoic, hydroxynonenoic) and then for C11:0 FAs (undecanoic, undecenoic, hydroxyundecanoic, hydroxyundecenoic).

**Figure 3:**
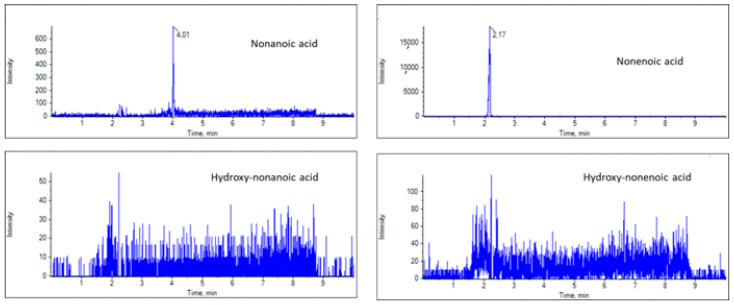
EICs of nonanoic, nonenoic, hydroxynonanoic and hydroxynonenoic acids in a representative RJ sample.

**Figure 4:**
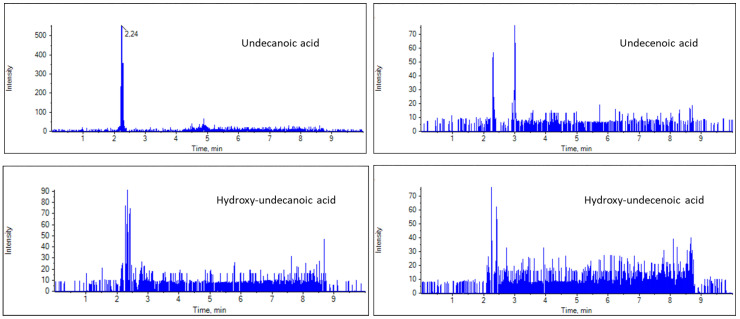
EICs of undecanoic, undecenoic, hydroxyundecanoic and hydroxyundecenoic acids in a representative RJ sample.

For the suspect LC-HRMS screening, the masses of the deprotonated ions corresponding to suspect FAs were calculated on the basis of the molecular formula, and EICs were created in MultiQuant 3.0.2. The exact masses of the deprotonated ions of nonanoic, nonenoic, hydroxynonanoic and hydroxynonenoic are 157.1234, 155.1078, 173.1183, 171.1027, respectively, while those of undecanoic, undecenoic, hydroxyundecanoic and hydroxyundecenoic are 185.1547, 183.1391, 201.1496 and 199.1340, respectively. The RJ samples were screened for these masses, and representative EICs of the C9 and C11 FAs are presented in Figure 3 and Figure 4. Nonanoic and nonenoic acids (Figure 3), as well as undecanoic acid (Figure 4,) were identified in good ion intensities and peak areas. However, hydroxynonanoic, hydroxynonenoic, undecenoic, hydroxyundecanoic and hydroxyundecenoic were not detected (minimum intensity threshold of 150) (Figure 3 and Figure 4).

The suspect analysis data are summarized in Table 2. The results showed high mass accuracy (less than 5 ppm) and acceptable isotopic fit values (MS Rank). The contents of FAs which were detected are expressed in relation to the content of palmitic acid, as calculated using the areas recorded by LC–HRMS. Among these suspect FAs, nonenoic acid was found to be the most predominant (71%, relative to C16:0, Table 2), Nonanoic and undecanoic acids were estimated as 3% and 1%, respectively.

**Figure 5:**
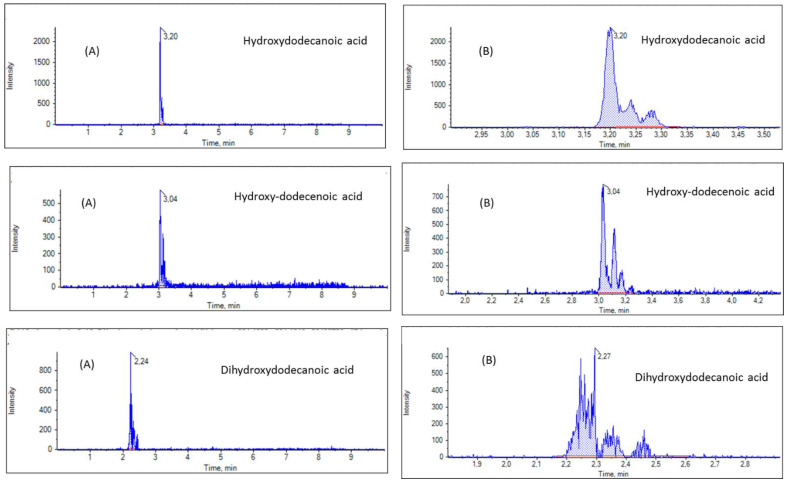
EICs of hydroxydodecanoic, hydroxydodecenoic and dihydroxydodecanoic acids (**A**) and zoom (**B**) in a representative RJ sample.

Next, we screened our RJ samples for masses corresponding to hydroxylated C12:0 FAs, more precisely to hydroxydodecanoic (or hydroxylauric acid). Hydroxydodecenoic and dihydroxydodecanoic acids and EICs of a representative sample are shown in Figure 5. The total peak area for hydroxydodecanoic acid ([M − H]^−^ exact mass 215.1653) indicated a 10% content relative to C16:0 (Table 2). Similarly, the total peak areas for hydroxydodecenoic acid ([M − H]^−^ exact mass 213.1496) and dihydroxydodecenoic acid ([M − H]^−^ exact mass 231.1602) indicated a 4% content relative to C16:0, for both of them (Table 2). However, as shown in Figure 5B, more than one peak was recorded for each one of hydroxydodecanoic, hydroxydodecenoic and dihydroxydodecanoic acids, indicating a mixture of isomers in all three cases. These findings are in accordance with the literature, reporting the presence of 10-, 11-, 12-hydroxydodecanoic acids [10,23,24], 10-, 11-, 12-hydroxy-2-dodecenoic acids [23,24], 3,12-, 10,12-, 10,11-, 3,11- and 3,10-dihydroxydodecanoic acids in RJ [23,24].

**Figure 6:**
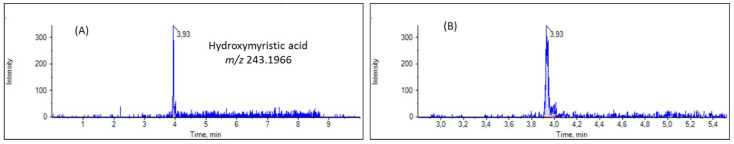
EICs of hydroxytetradecanoic acid (**A**) and zoom (**B**) in a representative RJ sample.

Furthermore, our RJ samples were screened by LC−HRMS for peaks corresponding to the exact mass of hydroxytetradecanoic acid (hydroxymyristic acid) ([M − H]^−^ exact mass 243.1966), and EIC for a representative sample is depicted in Figure 6. Two peaks were recorded, indicating the presence of two regio-isomers.

**Figure 7:**
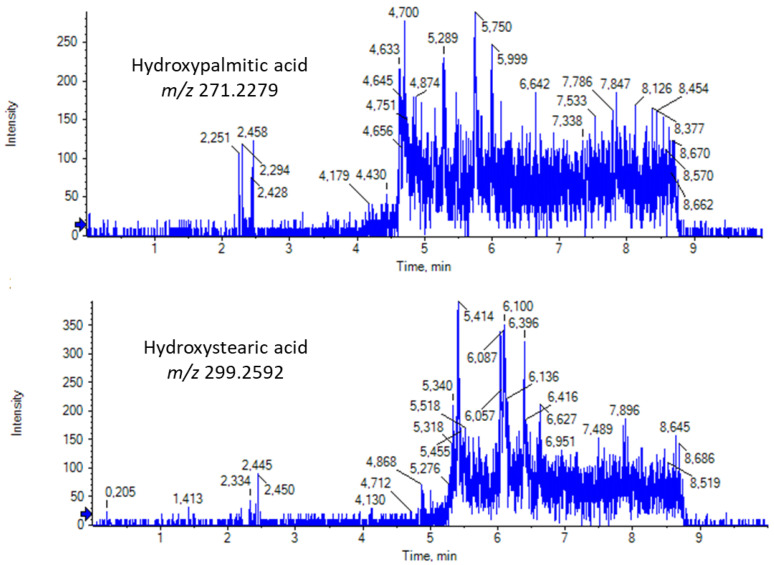
EICs of hydroxypalmitic and hydroxystearic acids in a representative RJ sample.

Finally, we screened our RJ samples for the existence of long-chain saturated hydroxy fatty acids (SHFAs). In 2020, we uncovered a previously unrecognized family of FAs, namely SHFAs, in milk and in human plasma, and we have demonstrated their beneficial biological effects [32]. This family consists of various regio-isomers, in particular, of palmitic and stearic acids, with the hydroxyl group at different positions of the long aliphatic chain. EICs of hydroxypalmitic acid ([M − H]^−^ exact mass 271.2279) and hydroxystearic acid ([M − H]^−^ exact mass 299.2592) in a representative RJ sample are presented in Figure 7. For the first time, the presence of a variety of regio-isomers of hydroxypalmitic and hydroxystearic acids is demonstrated in RJ samples. This is an interesting, novel finding, because such SHFAs may contribute to the bioactivities of RJ. Further investigation is needed to understand which peak corresponds to which regio-isomer.

### 3.4. Discussion

Studies on the common FAs (long-chain saturated and unsaturated FAs) in RJ samples are very limited. Investigating the lipidomic profile of RJ from different botanical origins, Yan et al. reported [27] that palmitic acid ranged from 14.47 to 20.88 mg/100 g of fresh RJ, stearic acid from 7.28 to 8.94 mg/100 g of fresh RJ, linoleic acid from 1.89 to 2.75 mg/100 g of fresh RJ and linolenic acid from 93.72 to 123.36 mg/100 g of fresh RJ. El-Guendouz et al. also identified common long-chain FAs in RJ samples from different origins [8]. In particular, by isolating RJ volatiles using hydrodistillation, they found that palmitic acid content ranged from 1.9 to 2.6% of RJ volatiles, oleic acid from 2.4 to 11.7% and linolenic acid from 2.2 to 50.3% [8]. It is worth noting that while Yan et al. found that nonanoic acid content ranged from 237.04 to 306.21 mg/100 g of fresh RJ, El-Guendouz et al. detected nonanoic acid in significantly lower quantities compared to other FAs, ranging from traces to 0.8% of RJ volatiles. In the same study, trans-2-nonenoic acid was detected in RJ samples ranging from traces to 1.0% of RJ volatiles [8].

In our study, we thoroughly analyzed a set of twenty long-chain FAs. Palmitic acid and stearic acid were found to be the most abundant among the saturated long-chain FAs, in accordance with Yan et al. [27] and El-Guendouz et al. [8]. However in our samples, mainly originating from Greece, oleic acid was found to be the most abundant unsaturated FA, followed by linoleic and linolenic acid, in contrast to Yan et al. [27], who reported linolenic acid as the most abundant (93.72 to 123.36 mg/100 g of fresh RJ and an absence of oleic acid. El-Guendouz et al. [8] detected linolenic acid at higher levels than oleic acid but no linoleic acid. As for the medium chain nonanoic and nonenoic acids, we estimated significantly higher quantities of nonenoic acid, in contrast to Yan et al. [27] but in accordance to El-Guendouz et al. [8].

The alterations of FA composition in various RJ samples observed in different studies may be attributed to the fact that various parameters, including botanical origin and environmental conditions, may affect the FA profiling significantly. Thus, depending on the origin of the RJ and the production conditions, the FA profiling may be essentially different.

Recently, we have uncovered the previously unrecognized family of SHFAs in milk, human plasma and yogurt [32,33,34]. We have shown that hydroxypalmitic and hydroxystearic acids exhibit growth inhibitory activities against various human cancer cell lines [32]. In addition, 7- and 9-hydroxystearic acids have been found to suppress β-cell apoptosis induced by pro-inflammatory cytokines, suggesting that they can be beneficial against autoimmune diseases, such as type 1 diabetes [32]. Thus, the novel finding of the present study that hydroxypalmitic and hydroxystearic acids are present in RJ samples is of notable interest and suggests further future investigation.

Overall, the present research may aid in uncovering low abundance long-chain FFAs, hydroxylated or not, that may contribute in the quality and the beneficial properties of RJ.

## 4. Conclusions

In conclusion, applying a targeted LC–HRMS method, we have studied the presence of twenty common long-chain FAs in seven RJ samples. The saturated palmitic and stearic acids, as well as the mono-unsaturated oleic acid, were found to be the predominant common FFAs. The high mass accuracy of LC–HRMS allowed the simultaneous use of a suspect approach to study in detail the presence of various C9 and C11 FAs as well as long-chain saturated hydroxy fatty acids in RJ samples. Regio-isomers of hydroxydodecanoic, hydroxydodecenoic and dihydroxydodecanoic acids were detected, in accordance with the literature. For the first time, the presence of a variety of regio-isomers of hydroxymyristic, hydroxypalmitic and hydroxystearic acids was demonstrated in RJ samples. All these derivatives of long –chain FAs may contribute to the bioactivities of RJ.

## Figures and Tables

**Table 1:** Contents of common long-chain FFAs in RJ samples (mg/100 g fresh RJ).

Free Fatty Acid	Royal Jelly Samples (*n* = 7), Triplicates			
	Minimum Value (mg/100 g RJ)	Maximum Value (mg/100 g RJ)	Mean Value ± SD (mg/100 g RJ)	*α*
C12:0	2.3	3.1	2.6± 0.3	***
C14:0	4.8	8.4	6.5± 0.5	***
C14:1	<LOQ	<LOQ	<LOQ	-
C15:0	<LOQ	<LOQ	<LOQ	-
C16:0	37.4	48.0	43.7 ± 3.9	***
C16:1	5.3	6.0	5.6 ± 0.2	***
C17:0	4.3	6.4	5.3 ± 0.7	***
C17:1	1.0	1.4	1.2 ± 0.1	***
C18:0	17.7	24.0	20.9 ± 2.1	***
C18:1	9.4	11.1	10.1 ± 0.6	***
C18:2	1.3	1.7	1.5 ± 0.1	***
C18:3	1.6	4.6	3.0 ± 0.5	***
C20:0	4.5	7.0	5.6 ± 0.9	***
C20:3	ND	ND	ND	-
C20:4	ND	ND	ND	-
C20:5	ND	ND	ND	-
C22:4	ND	ND	ND	-
C22:5	ND	ND	ND	-
C22:6	4.6	7.0	5.8 ± 0.9	***
C24:0	8.5	10.6	9.2 ± 0.6	***

<LOQ: lower of limit of quantification; SD: standard deviation; ND: not detected; α: level of significance; *** *p* < 0.001.

**Table 2:** Contents of suspect FFAs in RJ samples relative to palmitic acid.

Compound	Theoretical Mass [M − H]^−^	Measured Mass [M − H]^−^	Mass Error (ppm)	Elemental Composition	MS Rank ^a^	Content Relative to C16:0 (%) ^b^
Nonanoic acid	157.1234	157.1230	2.6	C_8_H_18_O_2_	1/1	3 ± 0.5
Nonenoic acid	155.1078	155.1077	0.6	C_9_H_16_O_2_	1/1	71 ± 9.1
Undecanoic acid	185.1547	185.1540	3.8	C_11_H_22_O2	1/1	1 ± 0.1
Hydroxydodecanoic acid	215.1653	215.1650	1.4	C_12_H_24_O_3_	1/1	10 ± 2.5
Hydroxydodecenoic acid	213.1496	213.1493	1.4	C_12_H_22_O_3_	1/1	4 ± 0.9
Dihydroxydodecanoic acid	231.1602	231.1600	0.9	C_12_H_24_O_4_	1/1	4 ± 0.9

^a^ MS Rank: The rank order based on the MS data. This uses a combination of mass accuracy and match to the theoretical isotope pattern. (1st of 1 hit); ^b^ by calculation of the peak area.

## Data Availability

Not applicable.

## Supplementary Materials

The following supporting information can be downloaded at https://www.mdpi.com/article/10.3390/biom13030424/s1, Table S1: List of analytes together with their exact masses [M − H]^−^, retention times (R_t_), limits of detection (LOD) and quantification (LOQ), accuracy (recovery %) and precision data (RSD %) in RJ samples. Figure S1: Sample preparation for the determination of free fatty acids in RJ samples. Figure S2: EICs of the analytes in a standard solution (500 ng/mL). Figure S3: EICs of each analyte in a representative RJ sample.
