# Supplementary material for: Targeted and Suspect Fatty Acid Profiling of Royal Jelly by Liquid Chromatography—High Resolution Mass Spectrometry
Source: Biomolecules. 2023 Feb 23;13(3):424. doi: 10.3390/biom13030424 (PMC10046394; doi:10.3390/biom13030424)
Supplement: Supplementary file 1 [file biomolecules-13-00424-s001.zip › biomolecules-2228215-supplementary.pdf]

Supplementary Material

Targeted and Suspect Fatty Acid Profiling of Royal Jelly by Liquid Chromatography-High Resolution Mass Spectrometry

Table of contents

1. **Table S1.** List of analytes together with their exact masses $[M-H]^-$, retention times (R_t), limits of detection (LOD) and quantification (LOQ), accuracy (recovery %) and precision data (RSD %) in RJ samples.
2. **Figure S1.** Sample preparation for the determination of free fatty acids in RJ samples.
3. **Figure S2.** EICs of the analytes in a standard solution (500 ng/mL).
4. **Figure S3.** EICs of the each analyte in a representative RJ sample.

Table S1. List of analytes together with their exact masses $[M-H]^-$, retention times (R_t), limits of detection (LOD) and quantification (LOQ), accuracy (recovery %) and precision data (RSD %) in RJ samples.

Reference compound	Exact mass $[M-H]^-$	R_t (min)	LOD (ng/mL) (29)	LOQ (ng/mL) (29)	%R	%RSD
Lauric acid (C12:0)	199.1704	5.2	0.6	1.8	90	11.87
Myristic acid (C14:0)	225.1850	5.9	0.6	1.8	96	10.68
Myristoleic acid	227.2017	5.4	0.6	1.8	88	8.15

(C14:1)						
Pentadecanoic acid (C15:0)	241.2173	6.2	0.8	2.4	86	7.33
Palmitic acid (C16:0)	255.2330	6.5	0.8	2.4	95	9.14
cis-9-Palmitoleic acid (C16:1)	253.2173	6.1	1.6	4.8	98	4.29
Margaric acid (C17:0)	269.2486	6.8	0.8	2.4	95	12.88
cis-10-Heptadecenoic acid (C17:1)	267.2330	6.3	0.8	2.4	98	8.67
Stearic acid (C18:0)	283.2643	7.1	1.0	3.0	96	10.07
Oleic acid (C18:1)	281.2486	6.7	0.8	2.4	93	5.63
Linoleic acid (C18:2)	279.2330	6.2	0.6	1.8	95	9.77
Linolenic acid (C18:3)	277.2173	5.8	0.6	1.8	100	6.57
Arachidic acid (C20:0)	311.2956	7.5	0.8	2.4	93	15.66
Dihomo-γ-linolenic acid (C20:3)	305.2486	6.6	0.6	1.8	93	5.21
Arachidonic acid (C20:4)	303.2330	6.2	0.6	1.8	95	7.41
cis-5,8,11,14,17-Eicosapentaenoic acid (C20:5)	301.2173	5.8	0.6	1.8	84	8.33
cis-7,10,13,16-docosatetraenoic acid (C22:4)	331.2643	6.8	0.8	2.4	87	4.32
cis-7,10,13,16,19-Docosapentaenoic acid (C22:5)	329.2486	6.1	0.4	1.2	86	4.87
cis-4,7,10,13,16,19-Docosahexaenoic acid (C22:6)	327.2330	6.1	0.4	1.2	92	5.01
Lignoceric acid	367.3582	8.2	0.6	1.8	96	8.71

(C24:0)						

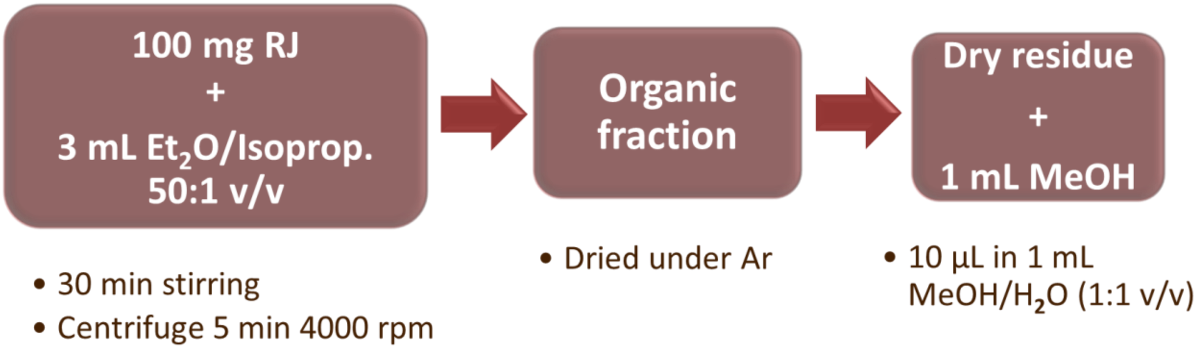

Figure S1. Sample preparation for the determination of free fatty acids in RJ samples.

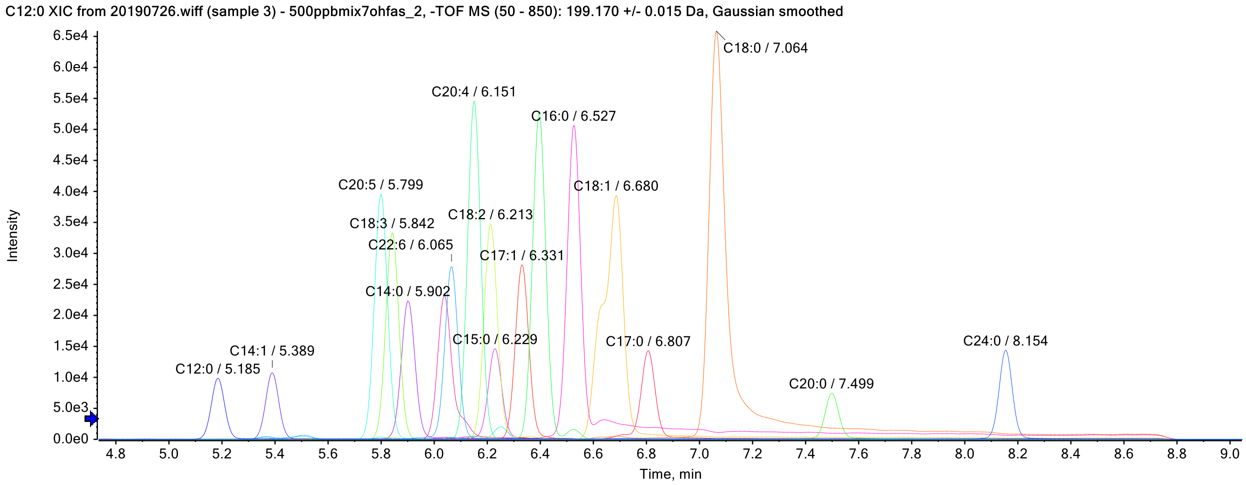

Figure S2. EICs of the analytes in a standard solution (500 ng/mL).

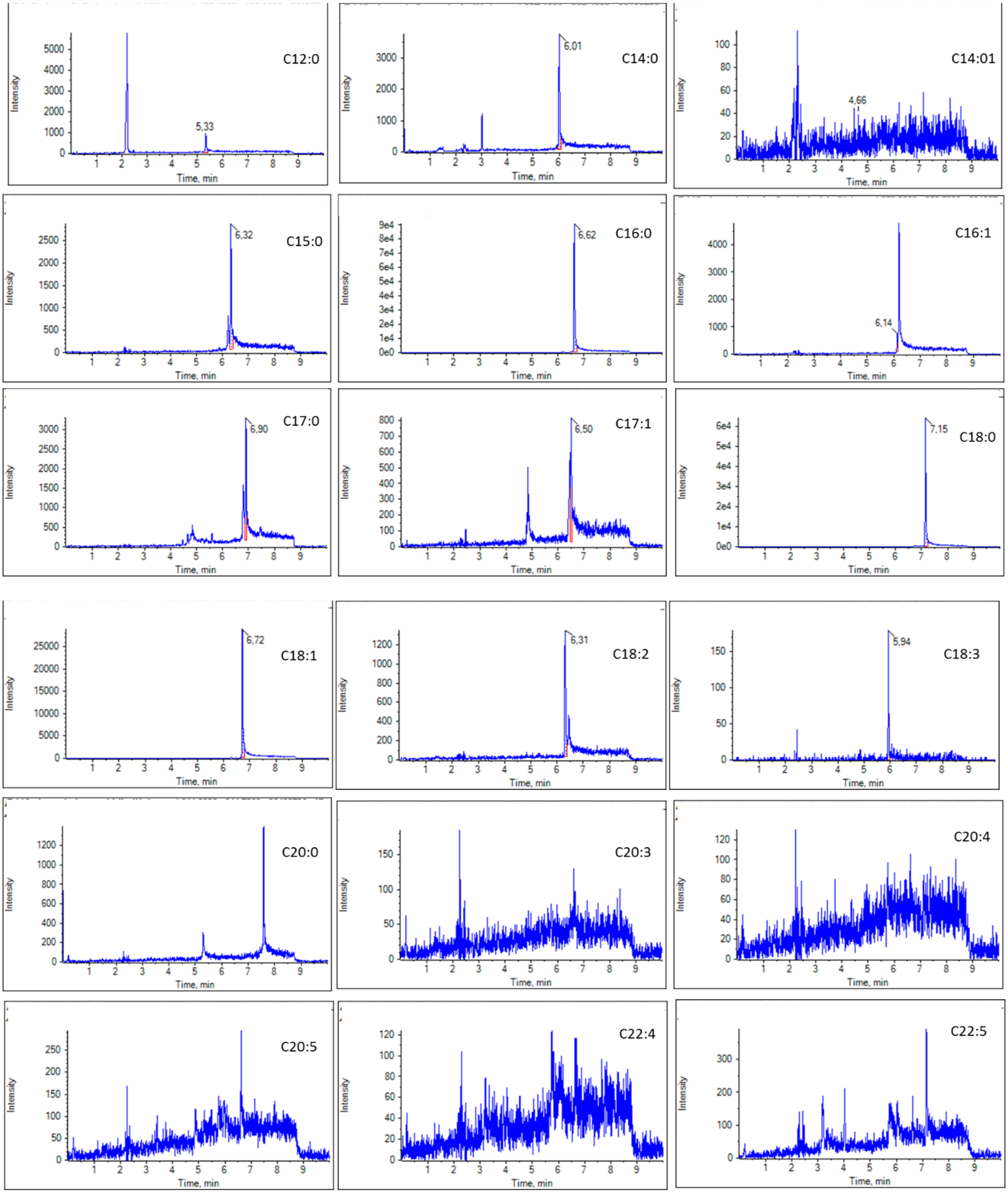
C12:0
5,33
C14:0
6,01
C14:01
4,66
C15:0
6,32
C16:0
6,62
C16:1
6,14
C17:0
6,90
C17:1
6,50
C18:0
7,15
C18:1
6,72
C18:2
6,31
C18:3
5,94
C20:0
C20:3
C20:4
C20:5
C22:4
C22:5
Intensity
Time, min

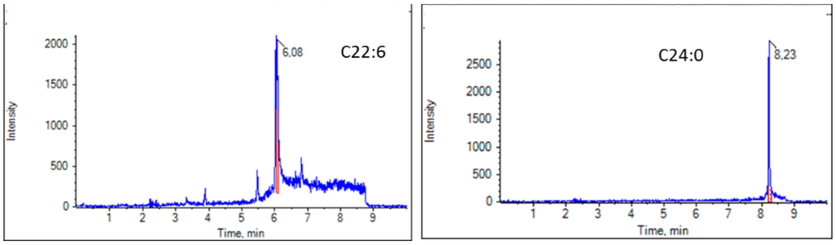

Figure S3. EICs of each analyte in a representative RJ sample.
